# Cellulose dissolution and gelation in NaOH(aq) under controlled CO_2_ atmosphere: supramolecular structure and flow properties†

**DOI:** 10.1039/d2gc02916b

**Published:** 2022-09-16

**Authors:** Guillermo Reyes, Alistair W. T. King, Tetyana V. Koso, Paavo A. Penttilä, Harri Kosonen, Orlando J. Rojas

**Affiliations:** Biobased Colloids and Materials, Department of Bioproducts and Biosystems, School of Chemical Engineering, Aalto University FI-00076 Espoo Finland guillermo.reyes@aalto.fi orlando.rojas@ubc.ca; VTT Technical Research Centre of Finland Ltd Tietotie 4e FI-02150 Espoo Finland; Materials Chemistry Division, Department of Chemistry, University of Helsinki FI-00560 Helsinki Finland; Biobased Materials Structure, Department of Bioproducts and Biosystems, Aalto University P.O. Box 16300 FI-00076 Aalto Finland; UPM Pulp Research and Innovations, UPM Paloasemantie 19 FI-53200 Lappeenranta Finland; Bioproducts Institute, Department of Chemical & Biological Engineering, Department of Chemistry and Department of Wood Science, 2360 East Mall, The University of British Columbia Vancouver BC V6T 1Z3 Canada

## Abstract

We investigate the interplay between cellulose crystallization and aggregation with interfibrillar interactions, shear forces, and the local changes in the medium's acidity. The latter is affected by the CO_2_ chemisorbed from the surrounding atmosphere, which, combined with shear forces, explain cellulose gelation. Herein, rheology, nuclear magnetic resonance (NMR), small and wide-angle X-ray scattering (SAXS/WAXS), and focused ion beam scanning electron microscopy (FIB-SEM) are combined to unveil the fundamental factors that limit cellulose gelation and maximize its dissolution in NaOH(aq). The obtained solutions are then proposed for developing green and environmentally friendly cellulose-based materials.

## Abstract 2

We investigate the interplay between cellulose crystallization and aggregation with interfibrillar interactions, shear forces, and the local changes in the medium's acidity.

## Graphical abstract

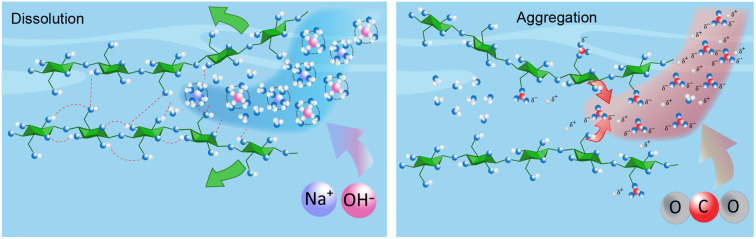

## Introduction

The forest products industries are exploring new dissolution and regeneration technologies to achieve high-performance materials with minimal environmental impacts.^1,2^ Industrially, four leading technologies have been used to dissolve cellulose. They include the Viscose and Lyocell methods, which are chemically intensive and present some environmental drawbacks.^3,4^ In this context, new generation Ionic Liquids (ILs) have gained importance and are expected to reach commercial adoption.^5,6^ Other solvent systems include aqueous solutions, such as Bemberg or cuprammonium hydroxide,^7^ and aqueous NaOH, already used in the mercerization process.^3,4^ The latter emerged in 1934 when NaOH_(aq)_ solutions were used to dissolve cellulose in a narrow window of conditions,^8–10^ eventually becoming attractive for cellulose regeneration due to the associated low environmental impact and cost.^4,11,12^ The dissolution of cellulose in sodium hydroxide solution has been associated with the interactions and hydrogen-bond disruption enabled by sodium and hydroxyl hydrated ions.^13–16^ Such phenomena occur at low temperatures (<−5 °C)^8–10^ and can be enhanced by freezing and thawing.^17^ However, sub-zero temperatures are required to improve dissolution and stability. Unfortunately, early gelation has so far inhibited alkali dissolution for large-scale adoption.^11,14^

Several routes have been proposed to enhance NaOH(aq) solvent dissolution capacity and stability. A recent example includes endoglucanases that hydrolyze cellulose and disrupt the intermolecular hydrogen-bonds, as shown in the Biocelsol™ process.^4,11,18^ The latter includes two additional aspects to improve the solubility and stability of the solution: (1) addition of ZnO, following the work of Davidson *et al.* in 1937 and, (2) freezing (−20 °C) and thawing.^9,17^ Other studies have reported urea and thiourea additives to improve solution stability (several days at room temperature).^18–28^ Recently, Liu *et al.*^15^ demonstrated, through molecular dynamic simulations, that urea's dissolving ability is mainly due to the high number density and hydrogen bonding of the molecules around the acetal oxygen atoms of cellulose (O1 and O5), preventing re-aggregation and extending solution stability. Other alternative solvent additives have been proposed,^14,29^ including amphiphilic polymers such as polyethylene glycol (PEG). Yan and Gao^30^ reported the dissolution of cellulose cotton linters at concentrations up to 13 wt% by using a NaOH(aq) (9 wt%) with 1 wt% PEG-2000. PEG amphiphilicity allowed interactions with the hydrophilic groups of cellulose, screening hydrophobic interactions and leading to cellulose chain entanglement in the aqueous system. The role of hydrophobic interactions has been discussed by Medronho *et al.*,^31^ who used an amphiphilic betaine derivative to delay gelation and increase the low critical dissolution temperature, as observed in aqueous NaOH solution.^32^ However, these types of additives are known to negatively impact the mechanical performance of regenerated cellulose due to the formation of porous structures derived from cellulose instability at the low pH of acid regeneration.^4,14,30^

To address the need for hydrophobic moieties and to allow for improved processing with aqueous NaOH, the ‘hydrophobic effect’^33^ is expected to contribute to the gelation of the cellulose–NaOH–H_2_O system. Indeed, Sobue *et al.*^32^ have charted the phase behavior of cellulose under different compositions. Just above the critical −5 °C dissolution/swelling temperature, cellulose is converted from cellulose I to ‘Na-Cell-IV’ (a hydrated form of cellulose II),^34,35^ allowing for regeneration as a cellulose II polymorph – an overall conversion from parallel to antiparallel chain orientation. Under the critical temperature, where the mixture is in a swollen or solution state, some interactions involving the hydrophobic surfaces of cellulose may lead to eventual antiparallel orientation, affecting the rheology. Thus, incorporating hydrophobic moieties, such as those in polyethylene glycol (PEG), is expected to reduce entanglement and lead to gelation *via* stabilization of the ‘hydrophobic’ surfaces. While this hypothesis is a reasonable one, one should consider that there may be a combination of other factors at play, both physical and chemical.

The present work introduces an approach to control the physicochemical environment used during cellulose dissolution, considering the freeze/thawing steps. We show the possibility of remarkably enhancing the dissolution of cellulose (tested here with microcrystalline cellulose) in an aqueous alkali solution at a high solids concentration, as high as 12 wt%.

In a CO_2_(g)-free atmosphere, the capacity of dissolved cellulose to absorb CO_2_ from the surrounding air is increased.^36,37^ Herein, we demonstrate that atmospheric conditions have a critical effect on the rheological behavior of cellulose solutions, a subject that has not been discussed so far. Furthermore, the crystallization and subsequent agglomeration, as evidenced by SAXS/WAXS, are shown to result from the molecular species formed during CO_2_(g) chemisorption (NMR and computational methods). Thus, this study reveals fundamental aspects that expand the possibility of NaOH-based systems for cellulose dissolution and regeneration at relevant scales, for instance, in fiber spinning.^38^

## Results and discussion

**Fig. 1:**
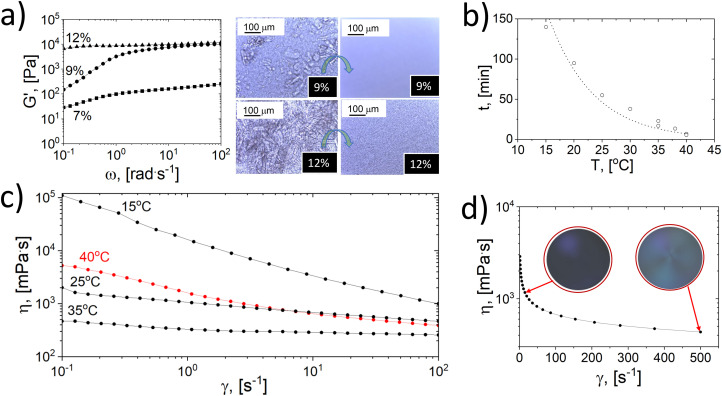
Gelation and flow properties of cellulose dissolved in NaOH(aq). (a) Effect of concentration on the elastic modulus and the corresponding microscope images before and after centrifugal thawing, the transition is indicated by the green arrow. (b) Master plot (gelation time), (c) viscosity and (d) shear thinning and birefringence (insets) of a cellulose solution at 7 wt% concentration.

We first realize the challenges in cellulose dissolution and regeneration due to the instabilities imposed by early gelation or polymer’ jamming’ under alkaline conditions.^14^ As such, we dissolve cellulose in a CO_2_(g)-depleted atmosphere followed by freezing and thawing under centrifugal forces (Fig. S1†). The dissolution and enhanced flow behavior are then confirmed by optical microscopy and rheometry (Fig. 1).

The elastic moduli (*G*′) of the samples (7, 9, and 12 wt% concentration) obtained after thawing are displayed in Fig. 1a, including optical microscopy images taken before and after thawing under centrifugal forces. As observed, the maximum elastic moduli measured at high frequency of cellulose solutions (12% and 9% concentration) plateau at *G*′ = 1 × 10^4^ Pa, indicating a gelated system. Moreover, with the increased concentration, the system reached terminal values of elastic modulus, complex and dynamic viscosities (Fig. S2†). The optical images (Fig. 1a) confirm a dissolved gel state at 12 wt% cellulose concentration, indicating a dissolution limit. Furthermore, a 13 wt% cellulose concentration undergoes gelling during dissolution (Fig. S3a†), with additional undissolved cellulose fibers. After freezing and thawing, the amount of undissolved fibers decreases marginally, and the system remains gelated (see Fig. S3b†).

The gelation of alkali-dissolved cellulose has been discussed,^39^ and time and temperature have been shown to influence molecular entanglement and crystallization.^16,39^

In this study, the kinetics of cellulose gelation below the linear viscoelastic region was followed by microscopy and rheology (Fig. 1b and S4†) and a master curve for a system with 7 wt% cellulose subjected to 1% strain (10 rad s^−1^) showed an exponential dependence between the time to gelation (*t*, min) and temperature (*T*, °C):^39^1
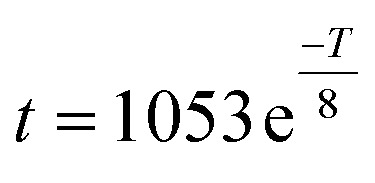


Eqn (1) was used to predict the gelation time under the tested conditions. For instance, gelation at 25 °C occurred in ∼1 h. Simultaneously, the apparent viscosity was reduced with temperature, accelerating the gelation process. Fig. 1c shows a non-gelated sample that underwent a sudden increase in viscosity at high temperatures (>40 °C) at the onset of gelation. We note that other factors besides time and temperature affect the gelation. This includes the freeze-thawing process,^9,17^ herein enhanced by centrifugation in a planetary movement (Fig. S1 and S5†).

Frozen samples thawed under centrifugal force at room temperature were less viscous than those thawed at rest for *ca.* 5 h (Fig. S5†). Shortening the thawing time delayed gelation, and, consequently, the dissolution capacity was improved after freezing and rapid thawing, as shown by microscopy imaging, Fig. 1a.

The cellulose system was subjected to high shear during centrifugation, eventually aligning cellulose fibrils.^40–42^ The cellulose system under high shear demonstrated shear thinning and birefringence (Fig. 1d), implying uniform and less entangled cellulose chains, impacting gelation stability. Fig. S6† shows that the initial liquid structure of a cellulose suspension is disturbed at high frequencies, recovering a liquid-like behavior (loss modulus, *G*′′ > elastic modulus, *G*′).

**Fig. 2:**
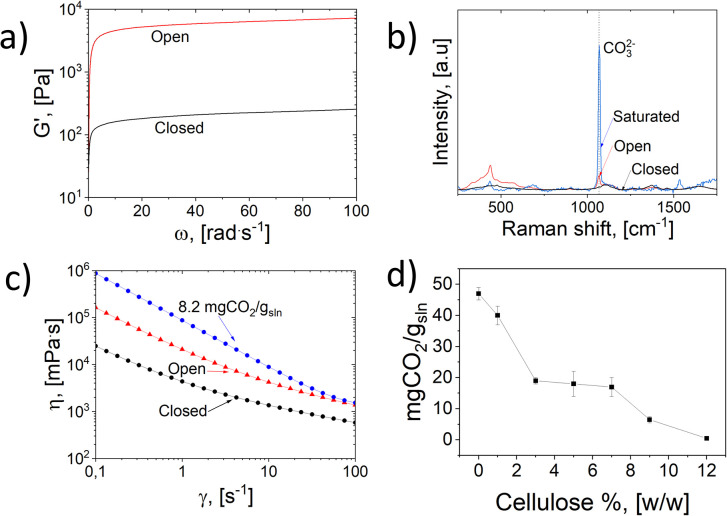
Gelation of dissolved cellulose during CO_2_(g) absorption at 7 wt% cellulose concentration: (a) effect on elastic modulus during contact with open atmosphere at room conditions during five days. (b) Raman identification of carbonate ions formed in the cellulose dissolution samples during gelation in open atmosphere. (c) Effect on viscosity of CO_2_(g) absorption compared to gelated samples (12 h, room conditions). (d) CO_2_(g) absorption capacity of alkali-dissolved cellulose.

The observed effects related to shear strain and oscillatory frequency are ascribed to the occurrence of a pre-gelation stage where interfibrillar interactions start to take place. Therefore, applying high shear stress at high frequency allows the recovery of a liquid-like behavior, destroying the metastable interactions before complete gelation. Hence, time, temperature, and flow forces are essential in gelation phenomena. Importantly, thawing in an open atmosphere led to solutions with a higher viscosity than those thawed in an air-tight environment (Fig. S5†). Rapid thawing in open-air conditions significantly affected the gelation process. This is related to the fact that cellulose dissolved in alkali absorbs and reacts with CO_2_(g),^37,43–45^ a subject that has not been widely acknowledged in relation to the gelation process. Fig. 2a indicates that contact of a solution with air during the gelation process (5 days) accelerated the entanglement of cellulose fibrils, increasing the elastic modulus by two orders of magnitude compared to samples prepared in air-tight conditions at room temperature.

The CO_2_(g) absorbed in the gelated sample was identified by Raman spectroscopy (see ESI†). Fig. 2b includes the main Raman shift band at 1064 cm^−1^ attributed to the non-degenerated symmetric stretching vibrational mode of carbonate (CO_3_)^2−^ ions.^46–49^ It is clear that the band intensity depended on the absorbed CO_2_(g). We noted that an aqueous cellulose solution (7 wt% concentration) absorbed up to 17 mgCO_2_ per g_solution_ until gelation (see ESI†), and exhibited the highest Raman intensity (blue line, Fig. 2b). By contrast, an air-tight sample did not show a Raman signal at this frequency (black line, Fig. 2b).

A sample saturated with CO_2_(g) formed a solid-like structured gel (Fig. S7†), becoming a rigid solid, which prevented any attempt to assess the flow properties. Moreover, fresh cellulose samples (7 wt%) prepared under conditions to achieve lower CO_2_(g) absorption from air, for instance set to 8.2 mgCO_2_ per g_solution_ (half the saturation value) were compared after gelation for 12 h in inert and open atmospheres (Fig. 2c and S8†). Compared to the samples prepared in sealed containers (12 h, 23 °C), the above gelated systems (12 h, 23 °C, 8.2 mgCO_2_ per g_solution_) showed one and two orders of magnitude higher viscosity, respectively. The elastic modulus revealed that the samples prepared in open-air and with added CO_2_(g) formed a stable gel. By contrast, the samples produced in the air-tight container formed a metastable gel with a reversible structure and a liquid-like behavior at high frequency (*ω* >10 rad s^−1^, Fig. S8†).

Previous studies,^37,43–45^ showed that CO_2_(g) absorption capacity drastically impacts cellulose gelation. However, the mechanism of this gelation and the effect of CO_2_(g) absorption as a function of cellulose concentration and viscosity are subjects that remain for elucidation.

In sum, the processability of dissolved cellulose was found to depend on the delayed gelation, which is affected by CO_2_(g) absorption (atmospheric conditions) and temperature. Fig. 2d presents the absorption of CO_2_(g) of cellulose samples (0–12 wt% concentration) until reaching saturation. The 7 wt% cellulose system absorbed 17(±3) mg CO_2_ per g_solution_. Meanwhile, the samples at 9 and 12 wt% concentration underwent instantaneous gelation and absorbed less CO_2_: 6.5 (±1) and 0.5 (±0.1) mg CO_2_ per g_solution_, respectively (Fig. 1a). We note that this process occurred at constant pH.^37^ As noted, the CO_2_(g) absorption capacity decreased with cellulose concentration, most likely due to the increased entanglement. Meanwhile, the high viscosity hinders CO_2_ accessibility and diffusion (Fig. S2†).

CO_2_(g) absorption by cellulose dissolved in NaOH(aq) solutions has been described as a chemisorption process, where the hydroxyl group on the C6 carbon of cellulose reacts to carbonate and carbonate ions, leading to a small pH drop (from 13.90 to 13.46).^36,37,45^ In aqueous media, carbonate ions and respective equilibrium species (carbonate/bicarbonate) are expected to increase attractive interactions and lead to gelation;^50^ this phenomenon is analogous to the protonation in acid media of the carboxylate groups present in TEMPO-oxidized cellulose nanofibrils, which reduce the electrostatic repulsion and lead to colloidal destabilization.^51^ Similarly, specific ions promote attractive interactions, establishing a micro-acidic environment and forming ligand complexes.^50^

We used NMR to study dissolved cellulose with and without CO_2_(g) (see ESI†). The results showed no significant changes in the cellulose structure except for the peaks of dissolved cellulose (Fig. S9†). This is in contrast to results obtained for cellulose carbonate structures.^37,43–45^

The amphiphilic nature of cellulose influences its interactions and forms highly solvated and coordinated ionic structures that disrupt hydrogen bonding. For instance, LiOH and NaOH alkali solutions dissolve cellulose due to the formation of hydration shells where the alkali ions form complexes with cellulose.^14^ Therein, the gelation kinetics depend on the increased hydrophobic interactions that promote cellulose precipitation/crystallization within a cross-linked network.^16^

**Fig. 3:**
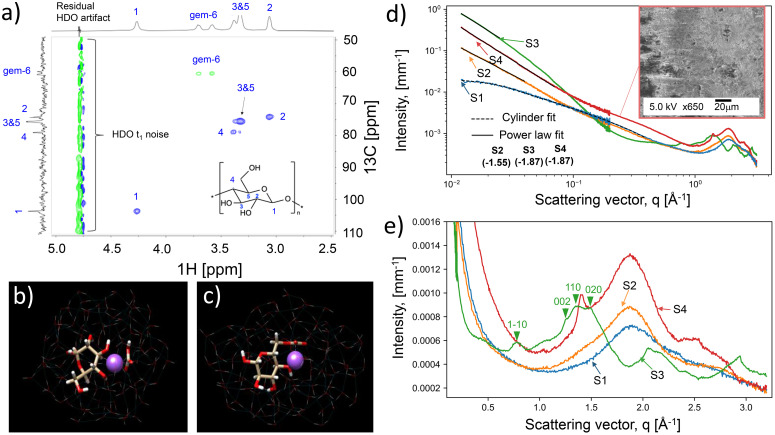
Chemical and molecular structure of cellulose during dissolution and CO_2_(g) absorption. (a) Assigned HSQC of 2.5 wt% MCC in D_2_O : H_2_O (50 : 50 wt%) NaOH solution, with a diffusion-edited 1H f2 trace. (b) Lowest energy conformer for geometry A obtained using the QCG method. H-bonds are shown in solid cyan lines and ionic bonds are shown in dotted purple lines. (c) Lowest energy conformer for Geometry B, obtained using the QCG method; H-bonds are shown in solid cyan lines and ionic bonds are shown in dotted purple lines. (d) SAXS intensity of all samples, showing a cylinder fit to S1 (radius 0.28 nm, length 16 nm) and power law fits to S2, S3 and S4 (power-law exponent in parentheses). Inset: FIB image of sample S4. (e) WAXS intensity of all samples, with the diffraction peaks in S3 indexed according to Na–cellulose IV (hydrate form of cellulose II).

The crystallization and aggregation caused by CO_2_(g) absorption were monitored through NMR and WAXS/SAXS experiments (see ESI†). Four samples were prepared at 2.5 wt% cellulose content: fresh (S1), sample aged for 7 days under air-tight conditions (S2), fresh sample aged in open room conditions for 7 days (S3), and a fresh sample with 15 mg CO_2_ per g_solution_ absorbed (S4) (Fig. S10†). S1 indicated cellulose backbone and high solvation *via* NMR correlations that were clearly assignable (^1^H–^13^C heteronuclear single-quantum correlation, HSQC, Fig. 3a). The full spin-system was traced – assignments were made through 2D HSQC-total correlation spectroscopy (TOCSY) NMR (Fig. S9†) and compared with the literature.^52^ The ^1^H spectra (Fig. S9a†) showed highly resolved signals confirming that cellulose was in a highly solvated state at 2.5 wt%. Comparison of the ^1^H spectrum, with the ^1^H water suppression enhanced through T_1_ effects (WET)-nuclear Overhauser effect spectroscopy (NOESY) and diffusion-edited ^1^H spectra identified minor heterolytic fragmentation, promoted under basic conditions (see ESI†).

The mechanism by which CO_2_ chemisorption leads to gelation was studied using the Quantum Cluster Growth (QCG) method,^53^ utilizing the Extended Tight Binding (xtb),^54^ and Conformer–Rotamer Ensemble Sampling Tool (CREST),^55^ software packages. The QCG method involves the automated explicit solvation of species, geometry minimization, conformer sampling using metadynamics,^56^ followed by GFN2-xTB,^57^ minimizations to give energetically accurate conformer-rotamer ensembles. The relative Gibbs free energies can be more accurately calculated by including the Gibbs–Shannon (conformational) entropy to the modified rigid-rotor-harmonic-oscillator (msRRHO) approximation, *i.e.*, yielding a more complete entropy calculation to include in the Gibbs energies. In our case, we solvated a glucose model with CO_2_, either in the form of a 6-OH carbonate or as a carbonate with one of the solvating water molecules – 100 water molecules were added to allow for several solvation layers – one Na^+^ ion was also included in each geometry, as counter-ion – all according to the following stoichiometries:Geometry A: Glucose − CO_3_^−^ + Na^+^ + 100 H_2_O (C_7_H_211_NaO_108_)Geometry B: Glucose + HCO_3_^−^ + Na^+^ + 99 H_2_O (C_7_H_211_NaO_108_)

Each geometry gave only 4–6 conformers (Table S1†) with a Boltzmann population of ≥0.1% each. The lowest conformers showed a strong interaction between the Na^+^, carbonates, and glucose OH (Fig. 3b and c). The calculated relative Gibbs free energies yielded only a 1.7 kcal mol^−1^ difference, which is rather small for a cluster weight of 2047 g mol^−1^. Thus, there is minimal energy difference between CO_2_ chemisorbed as a glucose-6-carbonate anion or as a hydrogen carbonate anion (combined with one of the solvating water molecules). Due to the large excess of water molecules during cellulose dissolution, the equilibrium will be away from cellulose carbonate formation. Therefore, the gelation and eventual precipitation of cellulose likely depends on simple acidification by CO_2_ chemisorption, leading to increased entanglement of cellulose and, less likely, involving a derivatized cellulose structure.

The CO_2_ adsorbed did not follow the dissolved cellulose content (Fig. 2d); this is a direct consequence of the chemical equilibrium involving CO_2_, in a NaOH-rich environment.^58^ At low cellulose concentrations, a large number of non-associated Na^+^ ions exist,^14^ which are counter-ions for equilibrating carbonate and bicarbonate.^58,59^ In contrast, at high cellulose concentration (≥5 wt%), NaOH ions are more strongly associated with cellulose. Hence, adsorbed CO_2_ scales strongly with the available reactive NaOH ions. This later observation is corroborated by the computational and NMR results, indicating that chemisorbed CO_2_ does not react with cellulose, at least at low CO_2_ loadings. Hence, once absorbed, CO_2_ primarily reacts with available NaOH ions.

With this as a starting point, SAXS results revealed that in the dissolved state, prior to gelation (sample S1), the cellulose chains were dissolved in a rather stiff conformation, as reported previously.^60^ Fitting a cylinder model^61^ to sample S1 (Fig. 3d) yielded a radius of 0.28 nm and length of 16 nm, indicating individually dissolved cellulose chains with straight segments of (approximately 30 glucose units).

The gelation showed up in the SAXS intensities as an increased contribution of low values of the scattering vector *q* (Fig. 3d).

A power law scattering contribution, corresponding to mass fractal dimensions of 1.5–1.9 at length scales above 10–20 nm, was found to increase in the order S2, S4, and S3; this is in line with the strongest gelation observed in the open-air conditions (sample S3 and Fig. S10†). Sample S3 exhibited a gelated, micron-scale network structure, supporting the nanoscale structure seen in freeze-dried samples by FIB-SEM. Identical structures were also observed on sample S4 (Fig. 3d, inset square). The WAXS data supported these interpretations, showing the emergence of diffraction peaks related to crystallization in samples S3 and S4 (Fig. 3e). In particular, the peaks of sample S3 can be indexed according to Na–cellulose IV (hydrate form of cellulose II) structure,^34,62,63^ confirming the non-derivatized presence of cellulose even in the presence of a saturated CO_2_ environment.

In general, accounting for dispersion, polarizability, and hydrogen-bonding interactions, the morphology of supramolecular cellulose hydrogels and their mechanical behavior can be modulated by their relative solubility.^64^ Furthermore, considering the ability of different ions to disrupt or stabilize hydrogen bonding in aqueous systems, for instance, following Hoffmeister's series according to their chaotropic or kosmotropic nature, it is possible to rationally control cellulose dissolution and gelation.^65^ Chaotropic agents prevent the interaction of non-polar regions, promoting solubility. Urea, thiourea, lithium hydroxide, sodium hydroxide, and polyethylene glycol (PEG) are chaotropic agents in alkali dissolved cellulose.^24,25,30,66^ By contrast, kosmotropic molecules cannot disrupt hydrogen bonds and, therefore, increase the hydrophobic effects in solution, promoting hydrophobic aggregation, entanglement, and further cellulose crystallization. The latter is the case of solvents such as sulfuric or hydrochloric acid,^50,51^ and according to the present results, it is also the case of absorbed CO_2_(g) from the air.

The formulation of aqueous alkali solvents for cellulose has included the addition of urea/thiourea, which affect the dissolution capacity and temperature/time stability through the formation of urea/thiourea hydroxyl complexes.^24,25,66^ Such molecules might also offer a buffering effect that influences the CO_2_(g) absorbed from the atmosphere.

In summary, the holding time, used as a variable to follow the gelation process, is related to the hydrophobic interchain interactions that promote cellulose's crystallization and gelation.^16^ Furthermore, according to the present results, a significant contribution to gelation can be assigned to media acidification by CO_2_ chemisorption – leading to increased interchain interactions.^37^ In this case, the shear forces appeared to facilitate the processability of the cellulose system.

## Conclusions

The present study reveals that adjusting time-dependent variables (CO_2_(g) chemisorption, ionic environment, temperature, and hydrophobic interactions) and processing shear forces allows an elegant control over the gelation phenomena, giving access to the production of cellulose solutions with a wide range of rheological properties, suitable for the preparation of different regenerated cellulose materials.

The present study advances the understanding of cellulose dissolution in alkali media. Further examination is needed to gain a fundamental understanding of the involved interactions and mechanisms.

## Author contributions

G. Reyes designed the study, conceived, and drafted the article. A. W. T. King and T. V. Koso performed the NMR experiments and performed the analysis of experimental data. P. A. Penttilä performed the analysis of the WAXS/SAXS experimental data. A. W. T. King carried out the computations and their interpretation. H. Kosonen and O. J. Rojas financed, co-supervised the research, discussed and proofread the manuscript. All authors contributed to writing the manuscript, and all authors read and approved the final version of the manuscript for submission.

## Conflicts of interest

There are no conflicts to declare.

## Supplementary Material

GC-024-D2GC02916B-s001
