# Supplementary material for: Cellulose dissolution and gelation in NaOH(aq) under controlled CO2 atmosphere: supramolecular structure and flow properties
Source: Green Chem. 2022 Sep 16;24(20):8029–35. doi: 10.1039/d2gc02916b (PMC9578387; doi:10.1039/d2gc02916b)
Supplement: GC-024-D2GC02916B-s001 [file GC-024-D2GC02916B-s001.pdf]

Electronic Supplementary Material (ESI) for Green Chemistry.
This journal is © The Royal Society of Chemistry 2022

Supporting Information

Cellulose dissolution and gelation in NaOH(aq) under controlled CO_2 atmosphere: supramolecular structure and flow properties

Guillermo Reyes,*[a] Alistair W. T. King,[b] Tetyana V. Koso,[c] Paavo A. Penttilä,[d] Harri Kosonen,[e] and Orlando J. Rojas *[a,f]

[a.] *Biobased Colloids and Materials, Department of Bioproducts and Biosystems, School of Chemical Engineering, Aalto University, FI-00076, Espoo, Finland.*
[b.] *VTT Technical Research Centre of Finland Ltd, P.O. Box 1000, FI-02044 VTT, Finland*
[c.] *Materials Chemistry Division, Department of Chemistry, University of Helsinki, FI-00560, Helsinki, Finland*
[d.] *Biobased Materials Structure, Department of Bioproducts and Biosystems, Aalto University, P.O. Box 16300, FI-00076 Aalto, Finland*
[e.] *UPM Pulp Research and Innovations, UPM, Paloasemantie 19, FI-53200 Lappeenranta, Finland.*
[f.] *Bioproducts Institute, Department of Chemical & Biological Engineering, Department of Chemistry and Department of Wood Science, 2360 East Mall, The University of British Columbia, Vancouver, BC V6T 1Z3, Canada.*

**To whom correspondence should be addressed. E-mail: guillermo.reyes@aalto.fi; orlando.rojas@ubc.ca*

Experimental section

Materials. Microcrystalline cellulose (MCC, Avicel PH-101, 50 µm particle size, DP 300) was purchased from Sigma-Aldrich (Merck KGaA, Darmstadt, Germany) and used with no additional modification. NaOH (99.6 % purity) from VWR Chemicals (Radnor, PA, USA) and ZnO (pro analysis) from Sigma Aldrich were used to prepare the cellulose solvent. All dissolutions were prepared in Milli-Q water (Millipore Corporation, Merck KGaA, resistivity 16 µS/cm).

Dissolution. MCC was dissolved in NaOH 2.3M maintaining a ZnO/NaOH mass ratio of 0.167 for different cellulose concentrations (0-12 wt%) according to the schematic diagram presented in **Figure S1**. The process included the following steps: first, the reagents, including cellulose and solvents, were degassed under vacuum (200 mbar, 30°C, 12h) to remove CO_2 and prevent its absorption from the air. The dissolution process was carried out in a reactor with an inert atmosphere of N_2 (Linde, Finland), whereby the whole air volume was replaced every minute. The dissolution took place in a vessel (-5°C, 4h, 300 rpm) with a cooling jacket that used as cooling agent a mixture of water/propylene glycol 1:1. After mixing, the dope was frozen (-17°C, 12h), following the conditions reported earlier.[1] The freezing step improves the NaOH hydrated shells' contact with the reactive hydroxyl groups of cellulose and enhances the dissolution. The solid dope was then thawed using a process that simultaneously homogenized and degassed the dope using a centrifuge with planetary movement (AR-250 mixer, Thinky Corporation, Tokyo, Japan). The solid dope was thawed under centrifugal forces (2000 rpm, 20 min), allowing the production of a transparent and fully dissolved dope (as observed by optical microscopy). The latter did not indicate the presence of air bubbles. The dope dissolution and gelation were monitored by optical light microscopy (DM 750, Leica Microsystems GmbH, Wetzlar, Germany) equipped with a camera ICC50HD (Leica).

CO_2 absorption. After thawing, CO_2 was bubbled into the cellulose dope at room temperature, using CO_2 gas (Linde, Finland) at P=1 bar. The CO_2 absorbed mass was measured on a high precision laboratory scale (MS-TS, Analytical Balance, readability 0.1mg, Mettler Toledo, Finland) until saturation (five independent repetitions).

Rheology. The shear rheology of the dissolved cellulose was monitored under steady and oscillatory modes using an Anton Paar Physica MCR 302 rheometer (Anton Paar GmbH, Graz, Austria). The rheometer was equipped with a Peltier hood H-PTD 200 for controlled temperature and humidity. A light source with a cross-polarized light and a camera were used to monitor the birefringence during the tests, which were carried out with a parallel plate geometry (25 mm diameter and 1 mm gap).

Confocal Raman Microscopy. Samples were measured by using a Confocal Raman microscope (Renishaw, inVia™ Qontor) with a laser at 532nm (100% power, exposure of 0.3 sec,100 accumulation, 100X lens, grating 830 l/mm). Samples intensities were the average of three different positions.

NMR. All NMR spectra were recorded on a Bruker Neo 600 MHz with a triple resonance probe-head. 2.5 wt% MCC samples were prepared as above, except for using 50 % v/v D_2O in purified H_2O. The probe temperature was 25 °C for all experiments. The diffusion-edited ^1H experiment (ledbpgp2s1d) used a diffusion delay of 0.2 s, a gradient pulse length of 2 ms at 90% intensity, a relaxation delay of 3s, an acquisition time of 0.5 s, and 32 transients. The HSQC (hsqcedetgpsisp2.3) and HSQC-TOCSY (hsqcdietgpsisp.2) experiments used 256 f1 increments (real and imaginary), 4 transients, an acquisition time of 0.064 s, a relaxation delay of 1.5 s and the HSQC-TOCSY has a TOCSY mixing time of 15 ms. The WET-NOESY water suppression experiment (noesywtpr1d) used 4 transients with an acquisition time of 4 s and a relaxation delay of 10 s. All data were initially processed in Topspin 4, and final images were prepared in Mestrenova 14.

Focused Ion Beam Scanning Electron Microscopy (FIB/SEM). The JIB-4700F (JEOL, Tokyo, Japan) with a hybrid conical objective lens, GENTLEBEAM™ (GB) mode, and an in-lens detector system were used to deliver a resolution of 1.6 nm at a low accelerating voltage (1 kV). We used an "in-lens Schottky-emission electron gun" that produced an electron beam with a maximum probe current of 300nA. For the FIB column, a high-current density Ga ion beam of up to 90nA maximum probe-current was employed for fast ion milling and processing of specimens. Before imaging, the samples were frozen in a cryogenic chamber. The images were analyzed using ImageJ.[2]

SAXS/WAXS. Small and wide X-ray scattering were measured using a bench beamline SAXS/WAXS device (Xeuss® 3.0 C, Xenocs SAS, Grenoble, France). The generator worked at 45 kV and 200 mA, with Cu Kα radiation. Samples at 2.5 wt% cellulose were measured in capillary tubes with a diameter of 1.5 mm. The sample **S1** consisted of a recently dissolved cellulose sample. The sample **S2** corresponded to the same **S1** sample but gelled/stored under inert conditions. Sample **S3** consisted of sample **S1** gelled in an open atmosphere for five days (23°C). The sample **S4** was prepared from a fresh sample with a known amount of absorbed CO_2 (15 $mgCO_2/g_{sln}$). **Figure S8** shows the appearance of the sample before SAXS/WAXS measurements. Background correction was done by subtracting the scattering of an empty sample holder, an empty capillary tube, and the solvent. The solvent contribution was manually scaled if needed to avoid negative intensities caused by sample heterogeneity and unavoidable air bubbles. The samples' final SAXS and WAXS intensities were merged and divided by the capillary thickness. The SAXS data of sample S1 was analyzed by fitting it with a model for randomly oriented cylinders,[3] using SasView 5.0.3 software.[4] For the other samples, the power law of the form $q^{-\alpha}$ was fitted with Python scripts to characterize the gel structure by mass fractal dimension α.[5]

Computational Results. Initial geometries were prepared using Avogadro 1.2.[6] The quantum cluster growth (QCG) method was applied using 99 or 100 water molecules with alpb water implicit solvation and the GFN2-xTB final minimization. Initial molecular dynamics simulation times were set to 50 *ps* initially. However, this did not seem to reach the global minimum, so it was extended to 1000 *ps* (~120 h of run time on 20 cores). The final 10 lowest energy conformers are shown in Table S1.

Table S1. Simulated geometries' conformer's lowest energies

Geometry A				Geometry B			
cluster	E_norm [Eh]	De [kcal]	p	cluster	E_norm [Eh]	De [kcal]	p
1	-563.01191	0.000	0.656	1	-563.01499	0	0.9052
2	-563.01094	0.603	0.237	2	-563.01286	1.3394	0.0944
3	-563.01012	1.119	0.099	3	-563.00692	5.0647	0.0002
4	-563.00768	2.651	0.008	4	-563.00593	5.6854	0.0001
5	-563.00457	4.604	0.000	5	-563.00554	5.9303	0
6	-563.00324	5.436	0.000	6	-563.00511	6.2001	0
7	-563.00234	6.001	0.000	7	-563.00455	6.5537	0
8	-563.00131	6.652	0.000	8	-563.00449	6.5909	0
9	-563.00116	6.745	0.000	9	-563.00431	6.7008	0
10	-563.00000	7.473	0.000	10	-563.00409	6.8427	0

Where *E_norm* is the final electronic energy in Hartrees, *De* is the final energy in kcal·mol^{-1}, and *p* is the Boltzmann population for each conformer. The final geometries are available upon request to the corresponding authors.

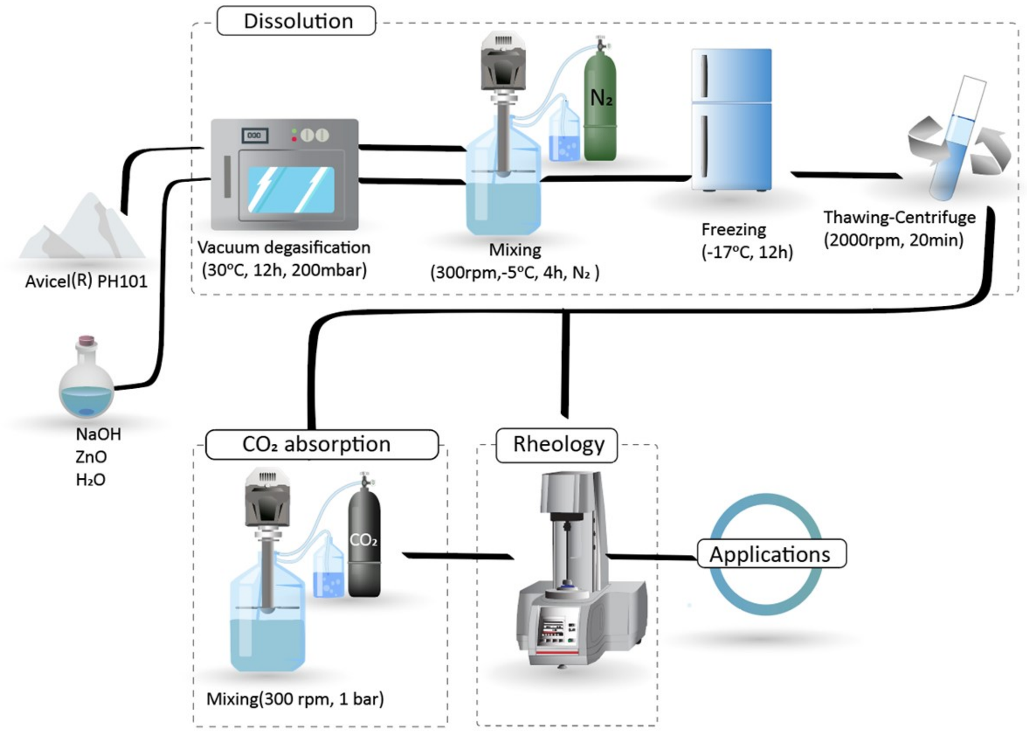

Figure S1-Experimental setup for cellulose dissolution in NaOH, including the freeze and thawing/centrifuge processes and CO_2(g) absorption stages.

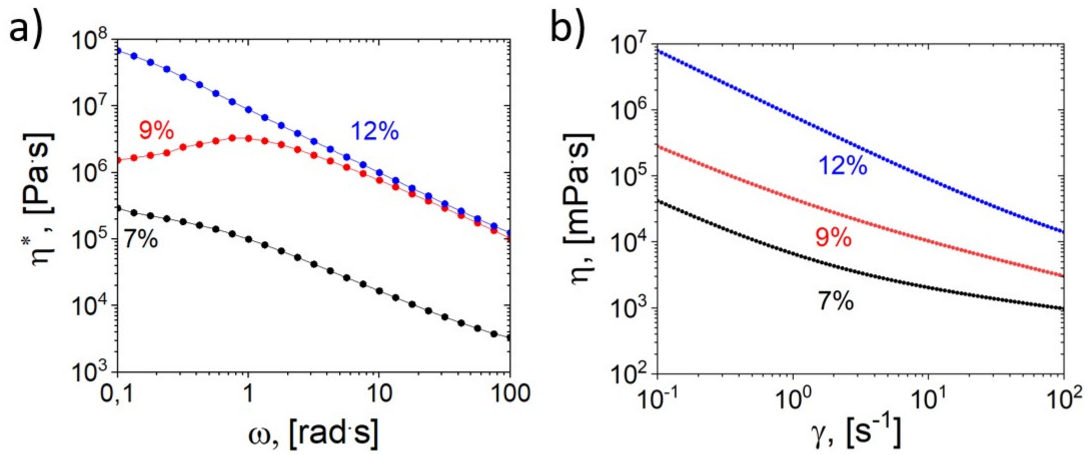

Figure S2-Complex and dynamic viscosities for recently thawed/centrifuged samples at different cellulose concentrations. a) Complex and b) dynamic viscosities.

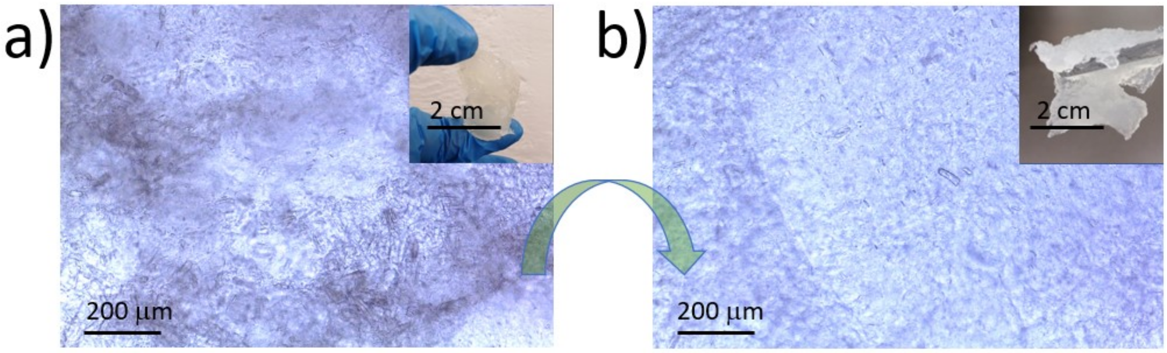

Figure S3-Optical microscope images of a 13 wt% cellulose dope, before and after centrifuge thawing. a) optical microscope of a 13 wt% dope during dissolution and gelation, inset image shows the physical aspect of the solid gel. b) optical microscope image after centrifuge thawing, inset image show a piece of the solid-like gel

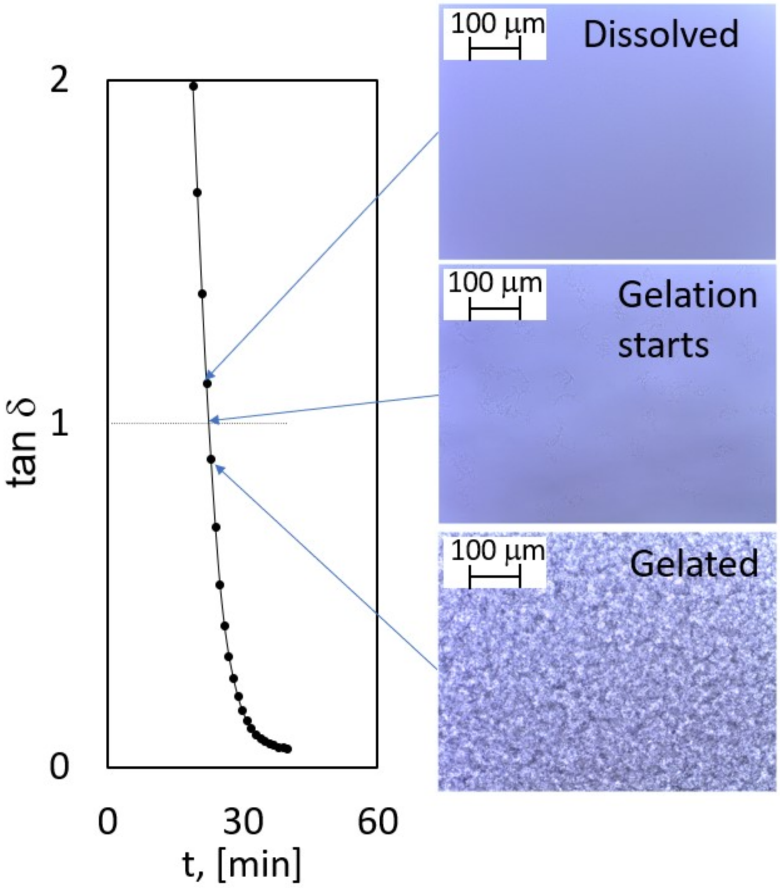

Figure S4-Phase angle and microscopy images during gelation at 30 °C degrees of 7 wt% cellulose, 1% strain and 10 rad·s^{-1}

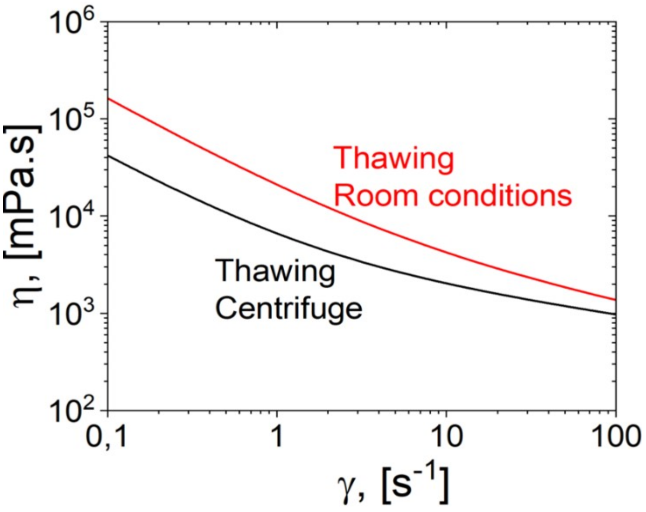

Figure S5- Dnamic viscosity and effect of thawing for a 7 wt% cellulose dope

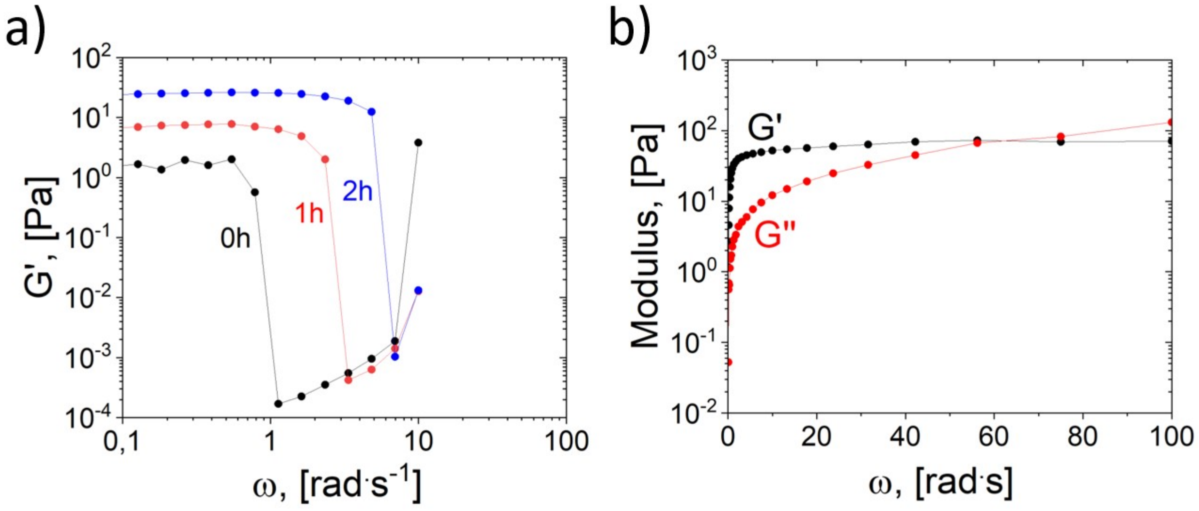

Figure S6-Elastic and loss moduli and effect of time and frequency for a 7 wt% cellulose dope in open atmosphere. a) Storage modulus variation with time. b) Elastic and loss modui registered after 7 hours

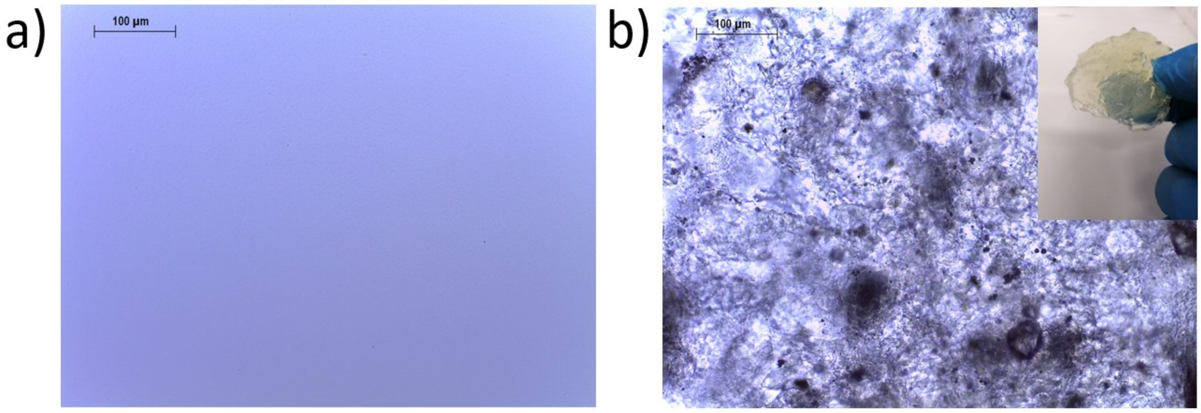

Figure S7-Optical microscope images of a 7 wt% cellulose dope, after thawing and after CO_2(g) saturation. a) optical microscope of a 7 wt% dope after thawing/centrifuge. b) optical microscope image after CO_2(g) saturation, inset image show a piece of the solid-like gel

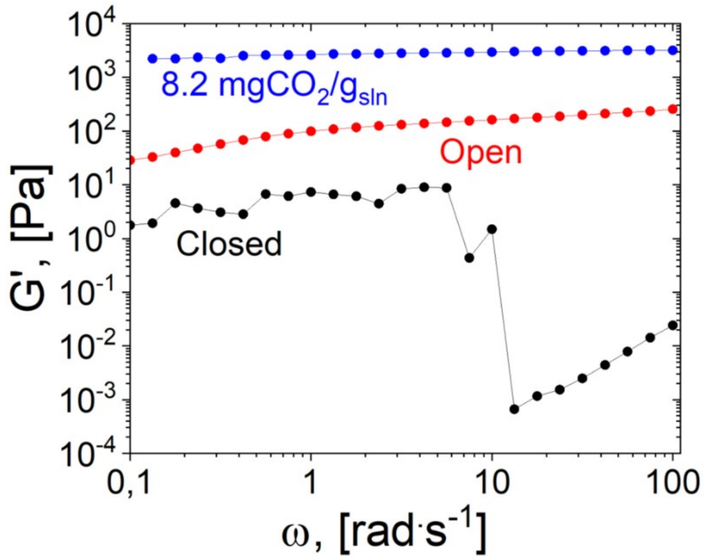

Figure S8-Elastic moduli and effect of atmosphere condition (air-tight and in contact with open air at room conditions) for a 7 wt% cellulose dope. Data for dope with a given amount of CO_2(g) absorbed.

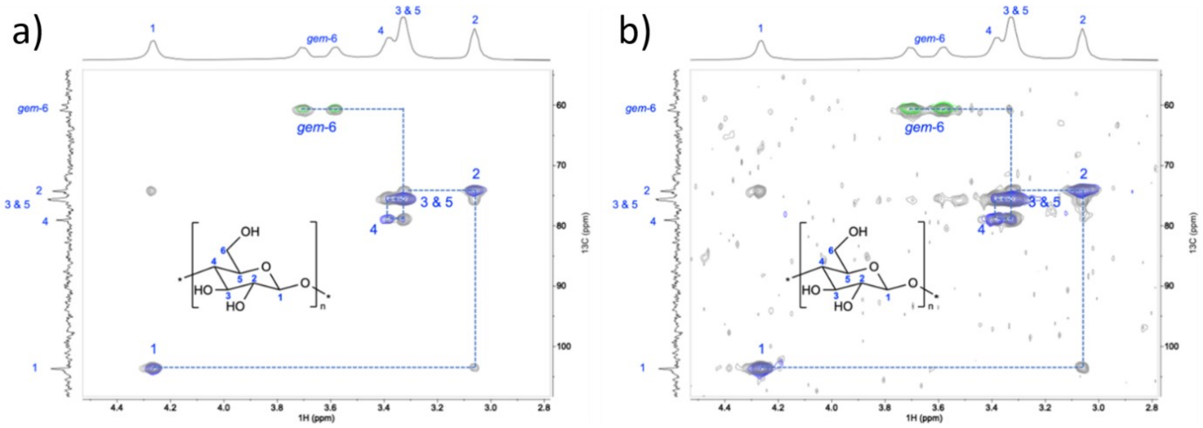

Figure S9-H NMR signal from dissolved cellulose in alkali conditions. a) fresh sample at 2.5 wt% cellulose. b) gelated samples at 2.5 wt% in open room conditions

References

1 S. Väisänen, R. Ajdary, M. Altgen, K. Nieminen, K. K. Kesari, J. Ruokolainen, O. J. Rojas and T. Vuorinen, *Cellulose*, , DOI:10.1007/s10570-020-03621-9.

2 National Institutes of Health, ImageJ, https://imagej.nih.gov/ij/index.html, (accessed 22 May

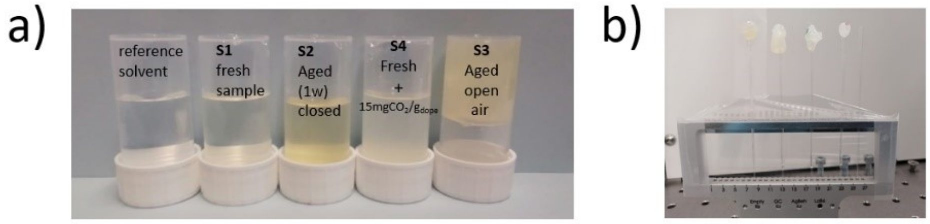

Figure S10-Samples appereance before SAXS/WAXS experiments. a) samples in bootle containers. b) samples located at the SAXS/WAXS sample holder

2017).

3 J. S. Pedersen, *Adv. Colloid Interface Sci.*, 1997, **70**, 171–210.

4 M. Doucet, J. H. Cho, G. Alina, Z. Attala, J. Bakker, W. Bouwman, P. Butler, K. Campbell, T. Cooper-Benun, C. Durniak, L. Forster, M. Gonzales, R. Heenan, A. Jackson, S. King, P. Kienzle, J. Krzywon, T. Nielsen, L. O'Driscoll, W. Potrzebowski, S. Prescott, R. Ferraz Leal, P. Rozycko, T. Snow and A. Washington, ,DOI:10.5281/ZENODO.3930098.

5 P. W. Schmidt, *J. Appl. Crystallogr.*, 1991, **24**, 414–435.

6 M. D. Hanwell, D. E. Curtis, D. C. Lonie, T. Vandermeersch, E. Zurek and G. R. Hutchison, *J. Cheminform.*, 2012, **4**, 17.
